# Systematic and benchmarking studies of pipelines for mammal WGBS data in the novel NGS platform

**DOI:** 10.1186/s12859-023-05163-w

**Published:** 2023-01-31

**Authors:** Qun-ting Lin, Wei Yang, Xin Zhang, Qi-gang Li, Yong-feng Liu, Qin Yan, Lei Sun

**Affiliations:** GeneMind Biosciences Company Limited, Shenzhen, China

**Keywords:** BSMAP, Bismark, BatMeth2, BS-Seeker2, BSBolt, GenoLab M, NovaSeq 6000, Epigenetics, 5-mC

## Abstract

**Background:**

Whole genome bisulfite sequencing (WGBS), possesses the aptitude to dissect methylation status at the nucleotide-level resolution of 5-methylcytosine (5-mC) on a genome-wide scale. It is a powerful technique for epigenome in various cell types, and tissues. As a recently established next-generation sequencing (NGS) platform, GenoLab M is a promising alternative platform. However, its comprehensive evaluation for WGBS has not been reported. We sequenced two bisulfite-converted mammal DNA in this research using our GenoLab M and NovaSeq 6000, respectively. Then, we systematically compared those data via four widely used WGBS tools (BSMAP, Bismark, BatMeth2, BS-Seeker2) and a new bisulfite-seq tool (BSBolt). We interrogated their computational time, genome depth and coverage, and evaluated their percentage of methylated Cs.

**Result:**

Here, benchmarking a combination of pre- and post-processing methods, we found that trimming improved the performance of mapping efficiency in eight datasets. The data from two platforms uncovered ~ 80% of CpG sites genome-wide in the human cell line. Those data sequenced by GenoLab M achieved a far lower proportion of duplicates (~ 5.5%). Among pipelines, BSMAP provided an intriguing representation of 5-mC distribution at CpG sites with 5-mC levels > ~ 78% in datasets from human cell lines, especially in the GenoLab M. BSMAP performed more advantages in running time, uniquely mapped reads percentages, genomic coverage, and quantitative accuracy. Finally, compared with the previous methylation pattern of human cell line and mouse tissue, we confirmed that the data from GenoLab M performed similar consistency and accuracy in methylation levels of CpG sites with that from NovaSeq 6000.

**Conclusion:**

Together we confirmed that GenoLab M was a qualified NGS platform for WGBS with high performance. Our results showed that BSMAP was the suitable pipeline that allowed for WGBS studies on the GenoLab M platform.

**Supplementary Information:**

The online version contains supplementary material available at 10.1186/s12859-023-05163-w.

## Introduction

DNA methylation at the fifth carbon position (C5) of cytosine is the conventional and pivotal epigenetic signature in the mammalian genome [1], namely 5 methylcytosines (5-mC). It can be reproduced through mitosis and is defined as the characteristic of cellular epigenetic memory or genomic imprinting [2]. Namely, the “persistent homeostasis” of 5-mC is extensive on the genome scale and it would be mediated and maintained by epigenetic switches according to cell status, tissue type, organisms state, and so on [3]. Functionally, 5-mC impacts embryonic development and several vital processes, such as the gene expression and chromatin remodeling [1, 4]. In terms of those functions, perturbations in methylation patterns, called failures of homeostasis of 5-mC, have been found to contribute to complicated disease etiology, including tumorigenesis, neurodegenerative disease, and neurological disorders [5–7]. Herein, the significance of this modification leads to a large and rapidly growing number of studies on itself [8].

Numerous experiments based on next-generation sequencing (NGS) could reveal DNA methylation status [9]. The current mainly approach adopts sodium bisulfite conversion to interrogate and quantify DNA methylation at nucleotide resolution [10], such as whole genome bisulfite sequencing (WGBS), and reduced representation bisulfite sequencing (RRBS). Due to the out-performance of efficiently detecting methylated cytosine at the whole genome scale and constructing robust whole-genome scale 5-mC methylation profiles [11], the WGBS assays is extensively used in the 5-mC analysis in mammalian genetics research nowadays. It is increasingly vital as a standard diagnostic tool in research and the medical setting for DNA methylome analysis [12]. Recently, Mary L. Stackpole et al. revealed the highly promising feature of specific individual methylation, used not only to detect cancer but also to locate its tissue of origin [13–15].

The development of new sequencers has been beneficial in reducing the high cost of WGBS, further rendering it possible to investigate various samples and resulting in rapidly increasing data for the usability of cancer detection (Additional file 1: Table S3). Currently, the principal sequencing instrument for WGBS is still the Illumina platform, such as NextSeq 550, and NovaSeq 6000 Sequencing System (NovaSeq). Those two sequencers use similar technologies and differ in fluorescence signals (4-color vs. 2-color) and corresponding basecall algorithms. In 2020, GeneMind released a new short-read sequencer, GenoLab M. This system offers both high- and mid-throughput flow cells, accommodating various sizes, throughputs, and turnaround times, for labs from large-scale to smaller (Additional file 1: Table S3). Presently, it demonstrated promising and robust performance in a wide variety of sequencing projects, such as LncRNA and transcriptome [16], WES and WGS [17], capable of calling multiple types of variants, including InDels and CNVs. However, its performance on WGBS has not yet been comprehensively assessed. GenoLab M platform for WGBS poses challenges, such as intact genomic representation, raw data quality, uniquely mapping ratio, data accuracy, the usability of suitable mapping tools, and potential bias introduced during sequencing, which is attractive for those wishing to integrate new sequencing data with data sequence on older platforms [18].

As for conventional methods for DNA library, due to bisulfite conversion of unmethylated cytidine residues after ligation of methylated adapters, many adapter-tagged DNA fragments are diminished and finally excluded in sequencing results, which results in viable reads drop [19]. The GenoLab M platform circumvents this limitation according to making bisulfite conversion take place before the ligation of sequencing adapters, namely as Post-Bisulfite Adaptor Tagging (PBAT) [20, 21]. This method reduces the DNA amount of sample required to at least 10 pg, avoids degradation of most DNA fragments during the bisulfite conversion, and produces highly diverse libraries as viable sequencing templates. This feature could enable more types of samples to be investigated, such as rare biopsies and cell lines [21].

Apart from intact genomic DNA fragmentation, the descriptions of data from the novel platform for methylation calls typically dissect other dimensions such as the properer pipelines, followed by preprocessing step, the proportion of mapped reads and uniquely mapped reads, the post-processing step, which is implemented after reading mapping and before methylation calls, genome-wide methylation levels, computational speed [22]. Those together bring us a more accurate interpretation of the bisulfite-seq dataset. In general, the critical point in silico analysis of WGBS is the alignment, which leads to specific challenges for the mapping tools due to the bisulfite conversion. There are two main approaches to mapping bisulfite-seq sequences: ‘wild card’ and ‘three letter’ mapping. Depending on the corresponding short-read alignment tool, those two approaches allow either gapped or ungapped mapping. Coupled with a proliferation of bisulfite-seq mapping tools [22], benchmarking studies have been implemented using real data downloaded and generated from platforms [23, 24]. According to those studies, in those dazzling tools, BSMAP [24–27], Bismark [22, 24, 27], BatMeth2 [28], and BS Seeker2 [27] outperform substantially all of the other pipelines based on different aligning algorithms, and corresponding information was listed in Table 1 For the mapping algorithm, Bowtie2 is much better for PE150 [29, 30], and Bowtie is much better for a shorter read [31]. Thus, in terms of our PE150 reads, we choose Bowtie2 as Bismark’s and BS Seeker2’s aligners (Table 1).

**Table 1:** Time-consuming from five software on human HEK293 samples in silico

Software/version	Character of building index	Indexing strategy	Alignment software^a^	Strategy	Final report?	Sample data (pair-end reads/Mb)	Time of building reference index	Alignment/h	Deduplication	Extraction of paired_read	Extraction of methylation	Total time
BSMAP v2.90	Building reference index in process of alignment	Hash tables	SOAP	Wild-card	No	105.675 *p* = 0.0013	0	10.478 *p* = 0.0014	24.5 (m) *p* = 0.0007	0	33 (m) *p* = 0.0001	11h26m
Bismark v0.22.3	Building multi-reference index at a time	FM index	bowtie2	3-letter	Yes		1h51m	7.025 *p* = 0.0015	35 (m) *p* = 0.0019	100 (m) *p* = 0.0010	7.15 (h) *p* = 0.0008	18h26m
BatMeth2	Building one reference index at a time	FM index	BatMeth2	3-letter	Yes		2h42m	13.07 *p* = 0.0017	113 (m) *p* = 0.0109			17h39m
BS Seeker2 v2.1.0	Building one reference index at a time	FM index	bowtie2	3-letter	No		3h17m	111.08 *p* = 0.0011	36 (m) *p* = 0.0003	0	30.85 (h) *p* = 5.236e-05	145h44m
BSBolt v1.4.8	Building one reference index at a time	FM index	BWA-MEM	3-letter with forked version of BWA-MEM and HTSLIB	No		2h18m	7.775 *p* = 0.0059	47 (m) *p* = 0.0025	0	7 (m) *p* = 0.0022	10h58m

^a^Short-read alignment pipeline used in this research for mapping bisulfite sequencing and extracting methylation pattern

^b^The parameters used in process was in default. The configuration of our operating system: Ubuntu v20.04 operating system, containing 2 physical CPUs, 48 logical CPUs, and 377G RAM size

Together, we obtained four human 293 (h293) and four mouse methylomes (representing two platforms from two individuals) and implemented five pipelines. To sum up, we confirmed the performance of GenoLab M on WGBS by parallel comparison with HiSeq Nova6000 on the well-characterized h293 and adult mouse liver tissue.

## Experimental procedures

### Sample preparation and genomic DNA isolation

Human cell line 293 was the kidney of a human embryo cell line 293 (HEK293), and was purchased from ATCC (VA, USA). Mouse liver cells was murine liver cell line NCTC1469. The mouse liver cells (< 25 mg) and HEK293 (< 5 × 10^6^) were acquired from Vazyme Biotech Co., Ltd., China, and GeneMind Biosciences. Genomic DNA (gDNA) from corresponding cells was isolated and extracted using FastPure^®^ Blood/Cell/Tissue/Bacteria DNA Isolation Mini Kit (Vazyme Biotech Co., Ltd., China) according to the manufacturer’s instructions. This kit performed an enzymatic approach to cell lysis, followed by protein precipitation and subsequent nucleic acid extraction, resulting in high purified, high-molecular-mass DNA. The amount of extracted DNA from two h293 samples and two mouse samples was preserved at -80℃ before library preparation [16].

### Bisulfite conversion, library preparation and sequencing

Bisulfite conversion of purified gDNA is performed using the EpiArt^®^ DNA Methylation Kit (EM101). With this method, bisulfite conversion was performed before adding sequencing adapters. Moreover, this kit employs thermal denaturation instead of traditional chemical denaturation. Meanwhile, it combines DNA denaturation and bisulfite conversion into one step and expedites the time of conversion reaction to less than 140 min. Finally, it resulted in high-yield of bisulfite-converted DNA (the conversion ratio is ≥ 99%, and the recovery efficiency is ≥ 80%).

Next, the bisulfite-treated DNA was purified and used to prepare the sequencing library using the EpiArt^®^ DNA Methylation Library Kit (NE103) according to the manufacturer’s instructions. In this procedure, sample-specific gDNA libraries were produced using unique dual indexes according to VAHTS Dual UMI UDI Adapters Set 1-Set 4 (N351 indexes which consist of 96 double-ended unique-dual-index (UDI) UMI adapters). Subsequently, those resulting sample-specific libraries were used to add P5/P7 adapters by PCR at the 5 and 3 ends, respectively, of the original DNA strand. Each index specific to a given sample was marked with a corresponding marker, such as mouse_s1, mouse_s2 (two replicates), or h293_s1, h293_s2 (two replicates) in the following in silico analysis. Each library was fragmented between the range of 200 bp and 500 bp using a 4150 Bioanalyzer DNA High Sensitivity kit (Agilent). Subsequently, each library was separated into two parallel libraries for two different sequencers and pooled. Libraries marked as Mouse_s1_NV and h293_s1_NV were pooled, and libraries marked as Mouse_s2_NV and h293_s2_NV were pooled, for NovaSeq platform. Libraries marked as Mouse_s1_GM and h293_s1_GM were pooled, and libraries marked as Mouse_s2_GM and h293_s2_GM were pooled, for GenoLab M platform. After library QC, pools were sequenced in PE150 mode on both sequencers, with the same 40% Phix spike-in [2] to improve the complexity of sequencing.

### Pre-processing

Raw reads in fastq format were identified by the index i5 sequences and divided into independently fastq files marked with corresponding sample names. Then, reads of each sample were checked for quality, including trimming adaptor, trimming low-quality bases, performing the quality score and read length filtering, by cutadapt [32] with the “-a GGGGGGGGGGGGX -a AGATCGGAAGAG -A AGATCGGAAGAG -A GGGGGGGGGGGGX -g CTCTTCCGATCT -G CTCTTCCGATCT” options to identify and trim adapter and poly-N, with the “-n 10 --max-n 0.05 -q 20,20 -u 10 -U 10 -m 30 -e 0.2” options to omit low-quality reads and produce clean fastq files. In terms of the additional tail sequence during ligation of the 3′ adapter and the insert size being smaller than the reading length, the bases from the header of the 5′ and the tail of 3′ need to be trimmed for more mapping efficiency. Non-trimmed and trimmed reads were quality-checked using FastQC v0.11.9 for adapter content, Q20, Q30, and QC quality checks. Dependent on the result of FastQC and BSMAP mapping, we choose 10 bp, 20 bp, 30 bp, 40 bp, and 50 bp to set parameters “-u” and “-U” as the trimmed length to detect a better mapping efficiency.

### Cross-platform m5C sequencing data analysis

Bisulfite-converted reference genome files, including human (GRCh38.p14) and mouse (mm10) were generated using Bowtie2 [30] on Bismark [33] and BS Seeker2 [34]. Alignment of QC-passed reads to the human and mouse reference files was performed with BSMAP v2.9.0 [35], Bismark v0.22.3 (Bowtie2), BatMeth2 [22], BS Seeker2 (Bowtie2), and BSBolt v1.4.8 (BWA-MEM) [36] respectively.

For all the approaches, we used default parameters. The post-precessing step is needed at Bismark, due to its particular requirement of uniquely mapping pair-end reads for subsequent methylation calling. The other process was implemented using the uniquely mapped reads for each aligner using corresponding post-processing programs, which could be used to estimate the percentage coverage and percentage methylation for all CpG sites on a genome-wide scale. All the alignment was implemented with 8 threads using a computational cluster with 377G RAM size and two physical CPUs containing 24 logical CPUs per physical CPU.

The Jaccard statistic, called the Jaccard similarity, could measure the similarity between two data sets to see which members are shared and distinct [37]. Here, we estimated the Jaccard statistic to reflect the similarity of the two sets uncovered by all different software [38]. All genomic characteristics were defined due to the GRCh38.p14 genomic annotation database acquired from NCBI. The promoter was defined as regions of ± 2 kb around transcription start locations [39]. The extent of overlap of methylated Cs and CpG from different pipelines was implemented using bedtools v2.27.1.

To verify the accuracy of the percentage methylation ratio of all chromosomes and specific genes by four sorts of software, we downloaded an average percentage of methyl-cytosines per chromosome in HEK-CT cells [40] and the percentage of methylation levels of *NSUN2* and *GNB1* genes from NCBI, belonging to GSM1254259 [40], GSM2467585 [41], GSM2425586 [42]. Subsequently, we compared previous studies on the methylation patterns of crucial locations of *GNB2* and *NSUN2* genes using house-in scripts and bedtools v2.27.1 [43] and visualized the methylated sites of those genomes using a custom R pipeline (Additional file 1).

## Results

### Comparison of read level and improving the mapping efficiency according to trimming

**Fig. 1:**
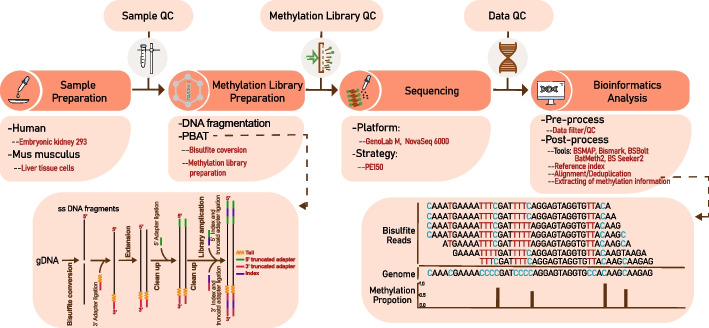
Schematic of the pipelines including key wet and dry labs

**Fig. 2:**
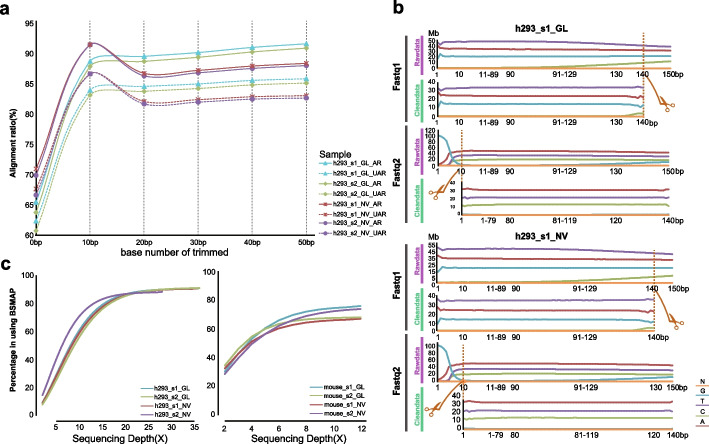
The effective number of trimmed bases in filtering process. **a** The whole-mapping ratio and unique-mapping ratio under different trimmed bases in filtering step. AR: all reads mapping ratio; UAR: unique all reads mapping ratio. **b** The site for trimming bases in read1 and read2. The orange dashed line and scissor are the cutting site for trimming. **c** The post-filtering depth and coverage of eight samples

Since the generation of high-quality WGBS data ultimately impacts the quantification and interpretation of Cs methylation levels, it is indispensable to monitor the raw data quality and interrogate the appropriate pre-processing step to cleanse data [1]. To avoid biased results by different sample preparation and library construction processes, we split the same library (Additional file 2: Fig. S1a and b) into two copies for two sequencing platforms. Finally, we obtained eight datasets as in Fig. 1. Based on an average of 321 bp length for two h293 libraries and 327 bp length for two mouse libraries, we generated 204.94 ± 43 (mean ± standard deviation (SD))million reads for the four h293 samples and 64.91 ± 12.01 million reads for the four mouse samples (Additional file 3: Table S1), of which the minimum value of Q30 was 90%. Even though high quality of raw data was obtained, due to the result of quality scores (Additional file 4: Fig. S2) and base distribution (Fig. 2b) using FastQC in the pre-processing step, we found that the first ten bases of read2 and the end ten bases of read1 showed relatively low quality. This phenomenon presumably resulted from the PBAT protocol, which suffered from a higher percentage of low-quality bases along the start of reads [39], or maybe because of the inadequacy of those sequencing instruments, requiring more detailed research in the follow-up research work [44]. Thus, we set various cutoffs, including 10 bp, 20 bp, 30 bp, 40 bp, and 50 bp (Fig. 2a) for the trimming stage and detected the mapping ratio of four h293 samples from the BSMAP mapping tool. The results determined that the proper number of the trimmed base from the 5′ of read2 and the end of read1 was 10 bp, which produced most of the viable amount of usable bases (of which, the minimum value of Q30 was up to 90.41%) and increased the mapping efficiency from 60 to 80% on unique mapping ratio (Fig. 2a; Additional file 4: Fig. S2). We loaded those cleansing datasets into BCREval [45] software to estimate the bisulfite conversion of those four libraries. The result showed that the conversion ratio was 96.685% for h293_s1, 95.745% for h293_s2, 93.97% for mouse_s1, and 93.41% for mouse_s2, respectively. That may result in false positive outcomes, such as a high level of non-CG methylation status. One particular challenge in carrying out WGBS is the base bias of the library. Therefore, we pooled ~ 40% PhiX spike-in, a substantial spike-in of DNA of a balanced base composition, to enhance sequencing quality. The result of base bias (Additional file 5: Fig. S3) showed the high-quality curated data. Besides, the relative decline of quality near the end of reads occurred on both sequencers. That may have been caused by the accumulation of phasing and prephasing errors in the PE150 sequencing. Based on this trimmed cutoff, we observed that the percentage of T base in whole reads from two platforms showed higher consistency and unbiasedness in PBAT step from another side (Additional file 6: Fig. S6a).

Using the QC-passed reads to align reference, we found that the four h293 data spread a balancing coverage, depth, and GC%, the same as mouse samples (Fig. 2c; Additional file 6: Fig. S6b). After the mapping stage using the BSMAP aligner, the data from GenoLab M reached a more robust depth (35x) [1] than that from NovaSeq, ranging from 28× to 33×, no matter in genome-wide or at chromosome-level (Fig. 2c; Additional file 6: Fig. S6c and d). The depth and coverage of data belonging to mouse samples showed consistency in the two platforms. The two replicates per platform and the average depth and coverage satisfied the modest sensitivity and specificity of standard WGBS [46].

### Comparison of those mapping performance and methylation conversion

**Fig. 3:**
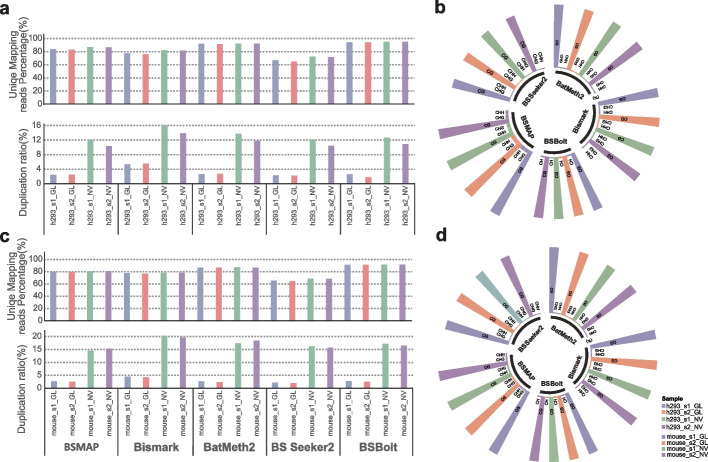
Alignment ratios and the methylated Cs’ ratio about CG/CHG/CHH. **a** The unique mapping ratio and duplication ratio of four h293 samples in four pipelines. **b** The radial histogram of the proportion of methylated CG/CHG/CHH in all identified Cs in four h293 samples. **c** The unique mapping ratio and duplication ratio of four mouse samples in four pipelines. **d** The radial histogram of the proportion of methylated CG/CHG/CHH in allidentified Cs in four mouse samples. For the result of BSBolt, the final proportions were only mCG and mCH (containing mCHH and mCHG). That is, there was no intermediate result for all CHH sites and CHG sites, preventing us from distinguishing the percentage of mGHG and mCHH

Apart from the data qualification, the mapping ratio was the second challenge mainly affected by computational methods and would impact the final methylation calls [27]. Herein, we then focused on comparing the mapping ratio and subsequently methylation conversion from the perspective of the platforms as well as data processing pipelines. From the platform’s perspective, it revealed that the alignment ratio was relatively higher in two h293 curated data from the NovaSeq platform, which was similar in four mouse curated data (Fig. 3a and c). The proportion of duplicated reads was much higher on NovaSeq (mean 14.94%) than that on GenoLab M (mean 3.05%) (Fig. 3a and c). In the comparison of software, we found that the alignment percentage for BSBolt was the highest and was superior to 94% on four h293 samples, and to 91% on four mouse samples. The following was BetMeth2, which could obtain at least 91% unique mapping reads on four h293 samples, and 86% unique mapping reads on four mouse samples. The performance of the BS Seeker2 aligner was the worst. The Bismark could find more duplicated reads on data from NovaSeq (mean 14.9%) than the other four sorts of software.

Additionally, multiple reads uncovering each methylated cytosine could be used as a readout of the fragmentation of the sequences within the sample that are methylated at that location, here represented as the methylation level of a specific cytosine. Therefore, we observed differences in methylation levels of the specific cytosine between the sequencing platforms and among the software of methylation calls. The detectable genome-wide Cs mainly contain non-CG context (CHG and CHH, where H = A, C, or T) and CG context (CpG sites). The methylated Cs percentile of curated data from the two platforms showed high conformity in both h293 and mouse samples. In the four sample reads (two h293 samples and two mouse samples) on GenoLab M, the methylated Cs of CpG (mCG) sites were significantly more than the methylated Cs of CHG (mCHG) and CHH (mCHH) (Fig. 3b and d). This trend of methylated Cs was similar in four samples on NovaSeq, indicating that at sites of non-CG methylation only a fraction of the surveyed genomes in those samples were methylated and data from the two platforms were entirely consistent. However, analysis of these loci from four software showed that BSMAP and BatMeth2 could reveal much more methylated Cs (~ 70–80%) than the remaining methods (~ 65%). This quantitative difference was much smaller than the unique alignment ratio.

### Assessment of the agreement between DNA methylation landscapes generated by pipelines versus that from two platforms

**Fig. 4:**
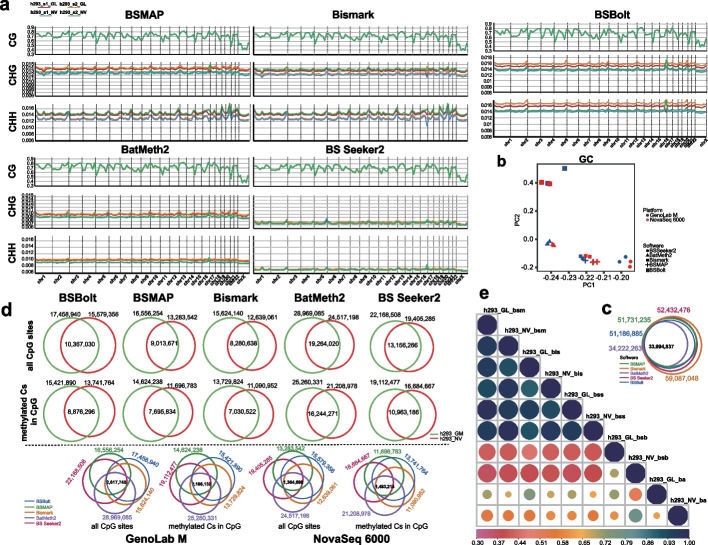
Comparing the methylated Cs profiles of two platforms from different software. **a** The methylation level of CG/CHG/CHH in each chromosome in four h293 samples from four sorts of software. **b** The PCA for datasets from four kinds of software. **c** The scanned CpG sites in four software. **d** The overlap methylated CpGs and all CpG sites in two replicates from two platform and four pipelines. h293_GM means this data is from GenoLab M platform, whereas h293_NV means that from NovaSeq 6000. **e** The correlationship of heatmap of all datasets from five pipelines and two platforms. bsm: BSMAP; bis: Bismark; bss: BS Beeker2; bsb: BSBolt; ba: BatMeth2

After strict curating the pre-processing and processing step, we evaluated the performance of two platforms for DNA methylation quantification and five processing pipelines for DNA methylation calls. In the genome of mammals, DNA methylation occurs mainly at cytosine followed by guanine, namely CG methylation (mCG) profile. Contrastingly, methylation status at cytosines followed by bases other than guanine is referred to as non-CG methylation (mCHG, mCHH, where H = A, C, or T) profiles. For two platforms, we focused on those three methylated Cs profiles (mCG, mCHG, mCHH) throughout each autosome and chrX. Of the detected Cs site in the CpG context, the density profile of mCG displayed consistency throughout each autosome and chrX from a global-scale view. On the contrary, the density profile of mCHG and mCHH showed more variations across each chromosome (Fig. 4a). Considering the testing mCs in all and the CpG context, we found that the GenoLab M platform calls considerably higher mCs relative to the NovaSeq platform (Fig. 4d). The common quantified mCpG sites accounted for ~ 50%.

For those processing pipelines, the profiled all Cs sites in the CpG context of the human genome showed that the Bismark could detect the most CG context, up to approximately 59 million, containing almost 99% CG context from the other four pipelines (Fig. 4c) and similar with the previous amount [4]. The density profile of mCpG was highly in agreement with data metrics from five processing pipelines. Conversely, the density profiles of methylated non-CG context from BSMAP and Bismark were similar and more noticeable than that from BSBolt, BatMeth2 and BSSeeker2. As a result of BSMAP, the density profile of methylated non-CG context on two platforms show more consistency from two replicates, which is vice versus Bismark. The BSSeeker2 uncovered the lowest density profiles of mCHG and mCHH among all software (Fig. 4a). For mCG loci, the correlation analysis of biological replicates and different approaches showed that BSMAP performed the best between replicates and platforms (Additional file 7: Fig. S4a).

To characterize the similarity of mCG and the clusters of those biological samples from all approaches, we performed the principal component analysis (PCA) using all mCG data. We generally observed a higher correlation between data from BSMAP and Bismark (Fig. 4b), a similar trend to the genomic-wide view of DNA methylation patterns (Fig. 4a). Among this result, two replicates on GenoLab M showed more similarity than two on NovaSeq from Bismark. Moreover, two replicates on NovaSeq showed more similarity than two on GenoLab M from BSMAP. For combined mCHG loci, the PCA analysis showed that the mCHG is more complicated and inconsistent among all pipelines and platforms (Additional file 7: Fig. S4b).

Hereafter, we combined two replicates to represent each platform and implemented the correlation analysis through pairwise comparison of mCG to excavate the concordance of data on two platforms from five approaches. We calculated the value of Jaccard similarity between pairs, and showed those correlation values via a heatmap plot (Fig. 4e). We observed that the data from BSMAP, BS-Seeker2, and Bismark showed more consistency, especially from BSMAP and BS-Seeker2. Furthermore, the data on two platforms from BSSeeker2 showed the most consistency. Intriguingly, that condition was observed in the analysis of identifying the common mCG sites and mCs sites according to the post-filtering mCG sites (depth per site ≥ 4) (Fig. 4d), which obtained the most common mCG sites and mCs sites up to 68%. However, a lower correlation was observed between mCG sites on two platforms from BatMeth2 and BSBolt (Fig. 4e). Due to the analysis of identifying the common mCG sites and mCs sites, we observed that of the methylcytosine detected in h293 on GenoLab M from BSMAP and BatMeth2, up to 88% were obtained in the CG context, and the total number of mCG sites was lower up to 84% on NovaSeq. That was a similar proportion between the two platforms from Bismark. For all pipelines, the common mC loci and mCG loci were lower in balance, and those were slightly increasing only for BSMAP and BSSeeker2, which was consistent with the result of correlation analysis (Fig. 4d and e). We further compared the paradigm of mCG methylation, which was validated by previous research [40], with our results of h293 from two platforms and four pipelines. The graph (Additional file 7: Fig. S4c) showed roughly similar methylation levels throughout each autosome and chrX.

Apart from the results’ concordance of the mappers based on the two platforms above, we simultaneously recorded the computational time on each procedure, including building reference index, pre-processing, alignment, post-processing, methylation calling, the convenience of use, and total time based on the same operating system configuration (Ubuntu v20.04 operating system, containing 2 physical CPUs, 48 logical CPUs, and 377G RAM size). We observed that BS-Seeker2 took the longest time in reference index, alignment, and methylation calls, whose time ranged from fourfold to tenfold longer than other pipelines. On the contrary, the BSBolt took the shortest time for the whole five procedures, which was only ~ 10 h on analysis of h293, following with BSMAP (~ 11 h) (Table 1; Additional file 8: Table S2).

### Consistency of methylated pattern of specific CG sites among data from two platforms and previous studies

**Fig. 5:**
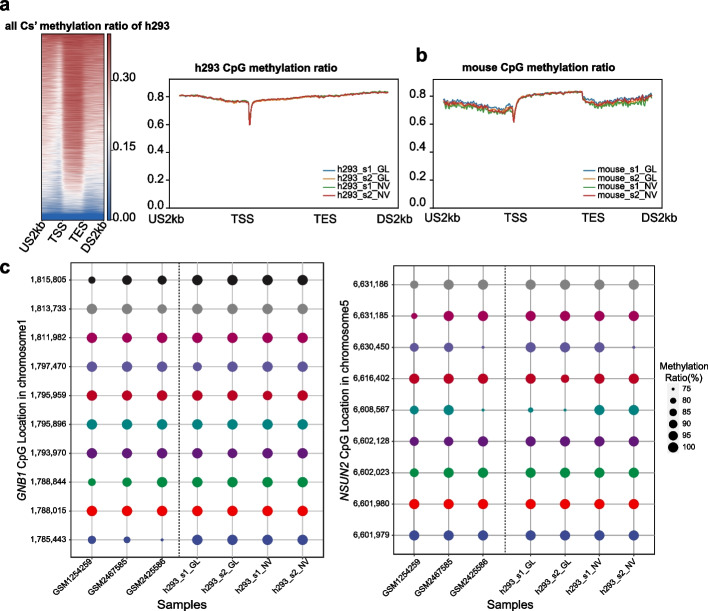
The distribution of methylated Cs in CpG about gene regions. **a** The methylation ratio of whole Cs and CpG sites in four h293 sampes from BSMAP. US2kb: upstream 2 kb; TSS: transcript start site; TES: transcript end site; DS2kb: downstream 2 kb. **b** The methylation ratio 0f CpG sites in four mouse samples. **c** The methylation levels of specific-site in *CNB1* and *NSUN2* genes. The x-axis means datasets from different origins; the y-axis means the specific-site in specific-gene

Despite the general concordance of the mapping outcomes, the results of the methylated calling demonstrated that BSMAP provided attractive robustness between replicates and platforms. Consequently, we further dissected the paradigm of mCs’ and mCGs’ methylation over genomic annotations from the result of BSMAP. Methylation rates plotted over GRCh38.p14 genomic annotation database was generated by aggregating all mCs methylation and mCG methylation fractions in percentile windows for 5000 bp upstream of the gene TSS, through the geneset, and the 5000 bp downstream of the gene TES. The result (Fig. 5a and b) revealed the same feature as the previous study [4].

We simultaneously compared the specific CpG of two genes in the human embryonic kidney cell line, which was validated by previous research [39], among our methylation results from BSMAP and those known results from NCBI [40–42]. The bubble plot showed highly consistent methylation patterns within all five datasets, indicating the accuracy of mCG sites on two platforms (Fig. 5c; Additional file 9: Fig. S5).

## Discussion

The GenoLab M platform is a new sequencing platform from GeneMind Biosciences, recently validated as a viable NGS sequencer in WES, WGS, RNA-seq, and lncRNA sequencing applications. The reproducibility of this sequencer and its concordance with main-stream sequencers on the mammalian genome has not been studied in WGBS, which is a crucial evaluation indicator of the newly established technology [26]. Hence, we have presented a benchmarking study about a human 293 cell line and mouse samples and anatomized concordance of those data from two dimensions, platform, and software, including publicly widely used Bisulfite-Seq software: BSMAP, Bismark, BatMeth2, BSBolt, and BSSeeker2 [25, 26].

As a determinant of the entire WGBS analysis workflow, the building of the bisulfite-Seq library is the first node needing attention for downstream methylation analysis. Those bisulfite-seq libraries are sequenced whether on GenoLab M or NovaSeq, the raw reads needed trimming from the start 10 bp of read2 and the end 10 bp of read1, which was attributed to the PBAT library preparation or other reasons requiring follow-up studies. Despite PBAT suffering from a higher percentage of low-quality bases along the start of reads [44], it could diminish the DNA amount of sample required down to 10 pg, improve degradation of most DNA fragments during the bisulfite conversion, and produce highly diverse libraries as effective sequencing templates [20]. In order to make up for that deficiency, trimming and quality filtering are indispensable. The cleansing data shows a better mapping ratio, GenoLab M especially exhibits more robust depth and coverage in h293 biological replicates (Additional file 6: Fig. S6c and d).

After that, we independently evaluated the mapping efficiency between the two platforms in each alignment algorithm. Comparing the platforms, we observed that the unique mapping ratio of bisulfite-seq reads on NovaSeq is higher by ~ 1%. Whereas, the percentage of duplication reads was much higher on NovaSeq than GenoLab M (14.94% vs. 3.05%), meaning that much more viable reads were obtained and used for the subsequent analysis procedures. This tendency was concordant with previous observations [47]. In the software comparison, the alignment percentage from BSBolt and BatMeth2 are both the highest, which is up to 91% in all data. The second best one is BSMAP, in which the M-bias plots show a higher consistency and lower CpG retention across the entire read length for both reads1 and reads2 after filtering (Additional file 4: Fig. S2: post-cleansing data). The duplication rate in Bismark is a mean of 2%, indicating that Bismark maybe contains a more strict cutoff for defining replication reads.

In the post-processing procedure using Bismark, we removed single-end unique mapping reads because of the request for methylation calls on only paired-end unique matching reads. To some extent, this discarding would result in the dropping some single-end sequencing reads containing informative methylated Cs sites. For example, our outcomes demonstrated that the Bismark tested the lowest methylated Cs sites (~ 65%) among pipelines. The subsequent in-house shell scripts and extra procedures increased the total analysis time. Though, the quantitative difference was much smaller than the unique alignment ratio. That trend of methylation conversion in h293 and mouse was undeviating with prevailing assumption [23], which demonstrated that mammalian genomic methylation levels are located almost entirely in the CG context, and a handful of studies have uncovered non-CG methylation in embryonic stem cells [48, 49]. Meanwhile, the methylation calling process shows differences in software. The BSMAP performs the best in running time and concordance of replicates and platforms. However, Bismark shows no obvious bias in analyzing the data of different platforms and could generate a final report for the whole methylation workflow, which is quite user-friendly. In addition, in terms of the lower depth in h293_s2_NV replicate, we decreased the filtering criteria of informative CpG sites to two and one supporting reads, and found that the ratio of common detecting informative CpG sites in two platforms was up to 77%, and 91.23%, respectively. That was observed in methylated CpG sites, up to 73%, and 84.56%, respectively, in two filtering conditions. Therefore, the relative lower depth of h293_s2_NV affected the potential informative CpG sites of the final result in some context, which suggested that the unique CpG sites from GenoLab M could be true positive sites.

Hereafter, we used the result from BSMAP to compare with known datasets, investigate the difference among those data and find that they show highly consistent methylation levels on CpG sites of *GNB* and *NSUN2* (Additional file 9: Fig. S5). We caution that the lack of more mammalian specimens and low sample number may skew the platform’s performance. Also, more uniform coverage depth on each sample may be required to determine the methylation detection accuracy across genome.

## Conclusion

In conclusion, we generated eight bisulfite-seq datasets based on two sequencing platforms. Based on these datasets, we compared choices of preprocessing steps, mapping algorithm, postprocessing methods, and methylation pattern estimation. In the preprocessing procedure, we found the appropriate data trimming protocol on read1 and corresponding read2. We validated that generally good concordance of those data on the two platforms and highlighted suitable mapping algorithms for each platform. Our study provides a standardized WGBS resource to benchmark new WGBS library preparation protocols and sequencing platforms.

## Supplementary Information

**Additional file 1: Table S3.** Property among GenoLab M, NextSeq X and NovaSeq 6000 platforms.

**Additional file 2: Fig S1.** Fragment size analysis of DNA isolated from medium of HEK293 cells and adult mouse liver cells. **a** The h293-s1 was the library for h293_s1_GL and h293_s1_NV. The h293-s2 was the library for h293_s2_GL and h293_s2_NV. **b** The mouse-s1 was the library for mouse_s1_GL and mouse_s1_NV. The mouse-s2 was the library for mouse_s2_GL and mouse_s2_NV.

**Additional file 3: Table S1.** Statistics of raw reads and clean reads.

**Additional file 4: Fig S2.** The M bias of h293 samples in pro-cleansing data and post-cleansing data.

**Additional file 5: Fig S3.** The A/T/G/C distribution of h293 samples and mouse samples in pro-cleansing data and post-cleansing data.

**Additional file 6: Fig S6.** The shortcoming for datasets. **a** The percentile of T base in reads. **b** The ratio of reads with different CG percentages. **c** The distritbution of depth according to chromosomes in four samples and two platforms. **d** The relationship betweent GC percentage and different depth reads. **e** The overlap CpG sites between two platforms in different filter parameters.

**Additional file 7: Fig S4.** The correlationship methylation ratio of chromosomes from different datasets. **a** The correlationship of sample data from four h293 samples and different software. **b** The PCA of methylation ratio of CHG/CHH from different software. **c** The methylation level of different chromosomes from different origins. bsm: BSMAP; bis: Bismark; bss: BS Seeker2; bsb: BSBolt; ba: BatMeth2.

**Additional file 8: Table S2.** Time-consuming from four softwares on mouse samples in silico.

**Additional file 9: Fig S5.** The methylation pattern of GNB1 genebody 40 loci.

## Data Availability

The data that support the findings of this study have been deposited into CNGB Sequence Archive (CNSA) [50] of China National GeneBank DataBase (CNGBdb) [51] with accession number CNP0003567.That contains the sequencing reads of all replicates in the fastq format, with reviewer’s link: http://db.cngb.org/cnsa/project/CNP0003567_dee1a02c/reviewlink/.

## Abbreviations

h293: Human embryonic kidney 293 cell line
WGBS: Whole genomic bisulfite sequencing
C5: The fifth carbon position (C5) of cytosine
5-mC: The 5 methylcytosine (5-mC)
mCG: Methylated Cs in CpG context
mCHG: Methylated Cs in CHG context
mCHH: Methylated Cs in CHH context
mouse: Adult mouse liver tissue
NGS: Next-generation sequencing
RRBS: Reduced representation bisulfite sequencing
LncRNA: Long non-coding RNA
WES: Whole exome sequencing
WGS: Whole genome sequencing
PBAT: Post-bisulfite adaptor tagging
gDNA: Genomic DNA
PCA: Principal component analysis
