# Supplementary material for: Systematic and benchmarking studies of pipelines for mammal WGBS data in the novel NGS platform
Source: BMC Bioinformatics. 2023 Jan 31;24:33. doi: 10.1186/s12859-023-05163-w (PMC9890740; doi:10.1186/s12859-023-05163-w)
Supplement: Supplementary file 2 — Additional file 2: Fig S1. Fragment size analysis of DNA isolated from medium of HEK293 cells and adult mouse liver cells. a The h293-s1 was the library for h293_s1_GL and h293_s1_NV. The h293-s2 was the library for h293_s2_GL and h293_s2_NV. b The mouse-s1 was the library for mouse_s1_GL and mouse_s1_NV. The mouse-s2 was the library for mouse_s2_GL and mouse_s2_NV. [file 12859_2023_5163_MOESM2_ESM.pdf]

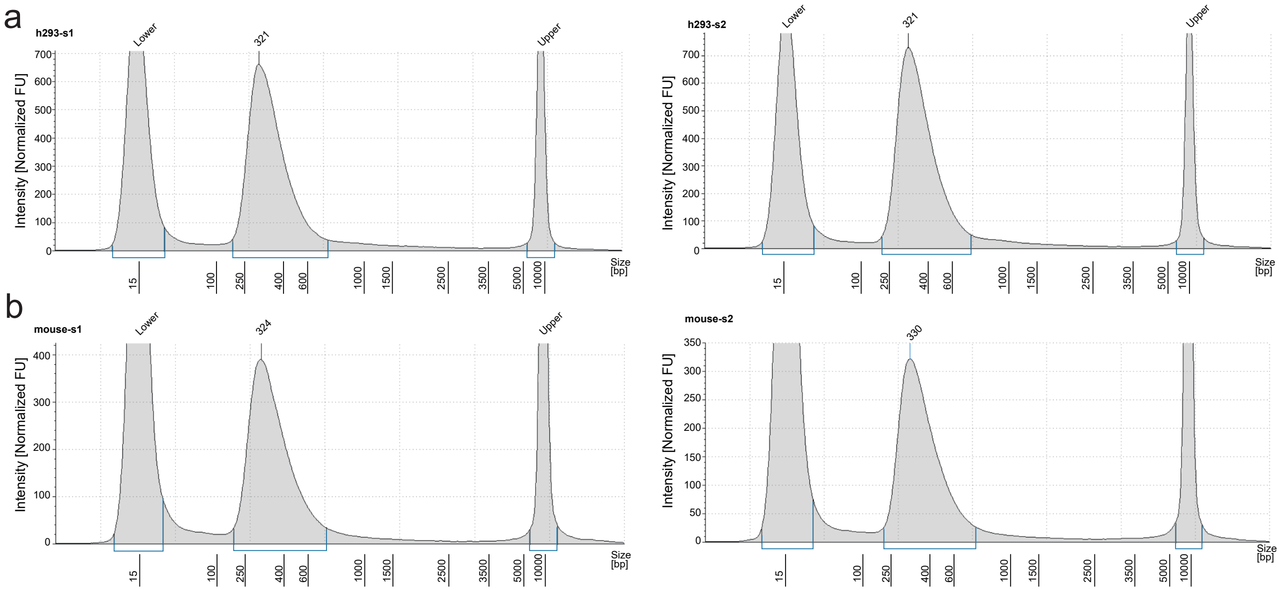

Supplementary Figure 1 Fragment size analysis of DNA isolated from medium of HEK293 cells and adult mouse liver cells. **a** The h293-s1 was the library for h293_s1_GL and h293_s1_NV. The h293-s2 was the library for h293_s2_GL and h293_s2_NV. **b** The mouse-s1 was the library for mouse_s1_GL and mouse_s1_NV. The mouse-s2 was the library for mouse_s2_GL and mouse_s2_NV.
