# Supplementary material for: Systematic and benchmarking studies of pipelines for mammal WGBS data in the novel NGS platform
Source: BMC Bioinformatics. 2023 Jan 31;24:33. doi: 10.1186/s12859-023-05163-w (PMC9890740; doi:10.1186/s12859-023-05163-w)
Supplement: Supplementary file 4 — Additional file 4: Fig S2. The M bias of h293 samples in pro-cleansing data and post-cleansing data. [file 12859_2023_5163_MOESM4_ESM.pdf]

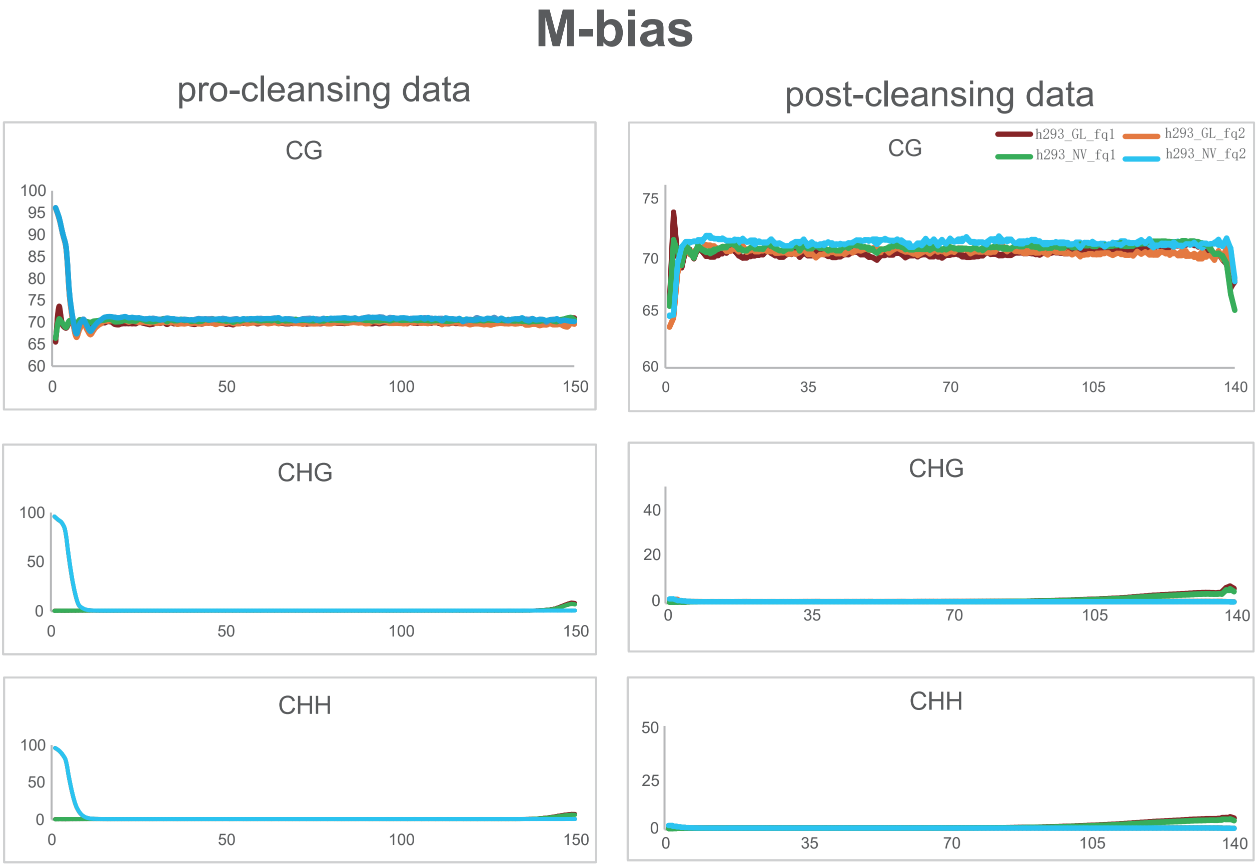

Supplementary Figure 2 The M bias of h293 samples in pro-cleansing data and post-cleansing data
