# Supplementary material for: Systematic and benchmarking studies of pipelines for mammal WGBS data in the novel NGS platform
Source: BMC Bioinformatics. 2023 Jan 31;24:33. doi: 10.1186/s12859-023-05163-w (PMC9890740; doi:10.1186/s12859-023-05163-w)
Supplement: Supplementary file 6 — Additional file 6: Fig S6. The shortcoming for datasets. a The percentile of T base in reads. b The ratio of reads with different CG percentages. c The distritbution of depth according to chromosomes in four samples and two platforms. d The relationship betweent GC percentage and different depth reads. e The overlap CpG sites between two platforms in different filter parameters. [file 12859_2023_5163_MOESM6_ESM.pdf]

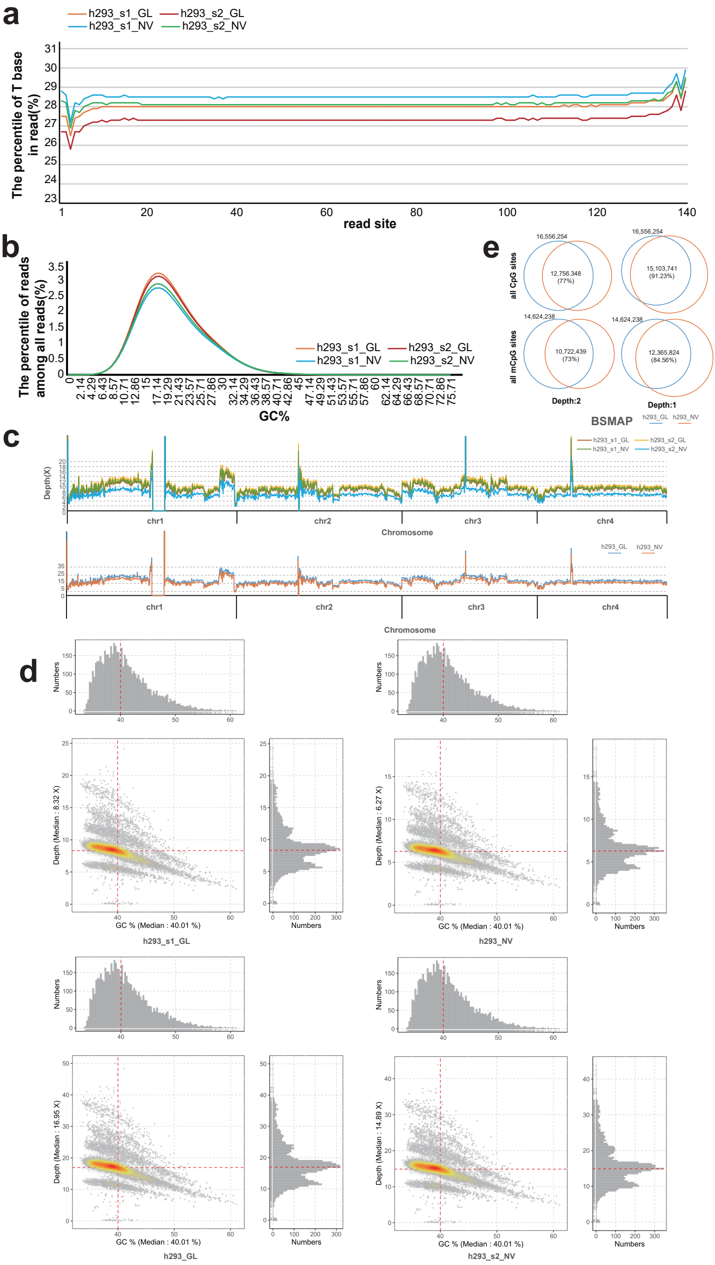

Supplementary Figure 6 The shortcoming for datasets. **a** The percentile of T base in reads. **b** The ratio of reads with different CG percentages. **c** The distritbution of depth according to chromosomes in four samples and two platforms. **d** The relationship betweent GC percentage and different depth reads. **e** The overlap CpG sites between two platforms in different filter parameters
