# Supplementary material for: Systematic and benchmarking studies of pipelines for mammal WGBS data in the novel NGS platform
Source: BMC Bioinformatics. 2023 Jan 31;24:33. doi: 10.1186/s12859-023-05163-w (PMC9890740; doi:10.1186/s12859-023-05163-w)
Supplement: Supplementary file 7 — Additional file 7: Fig S4. The correlationship methylation ratio of chromosomes from different datasets. a The correlationship of sample data from four h293 samples and different software. b The PCA of methylation ratio of CHG/CHH from different software. c The methylation level of different chromosomes from different origins. bsm: BSMAP; bis: Bismark; bss: BS Seeker2; bsb: BSBolt; ba: BatMeth2. [file 12859_2023_5163_MOESM7_ESM.pdf]

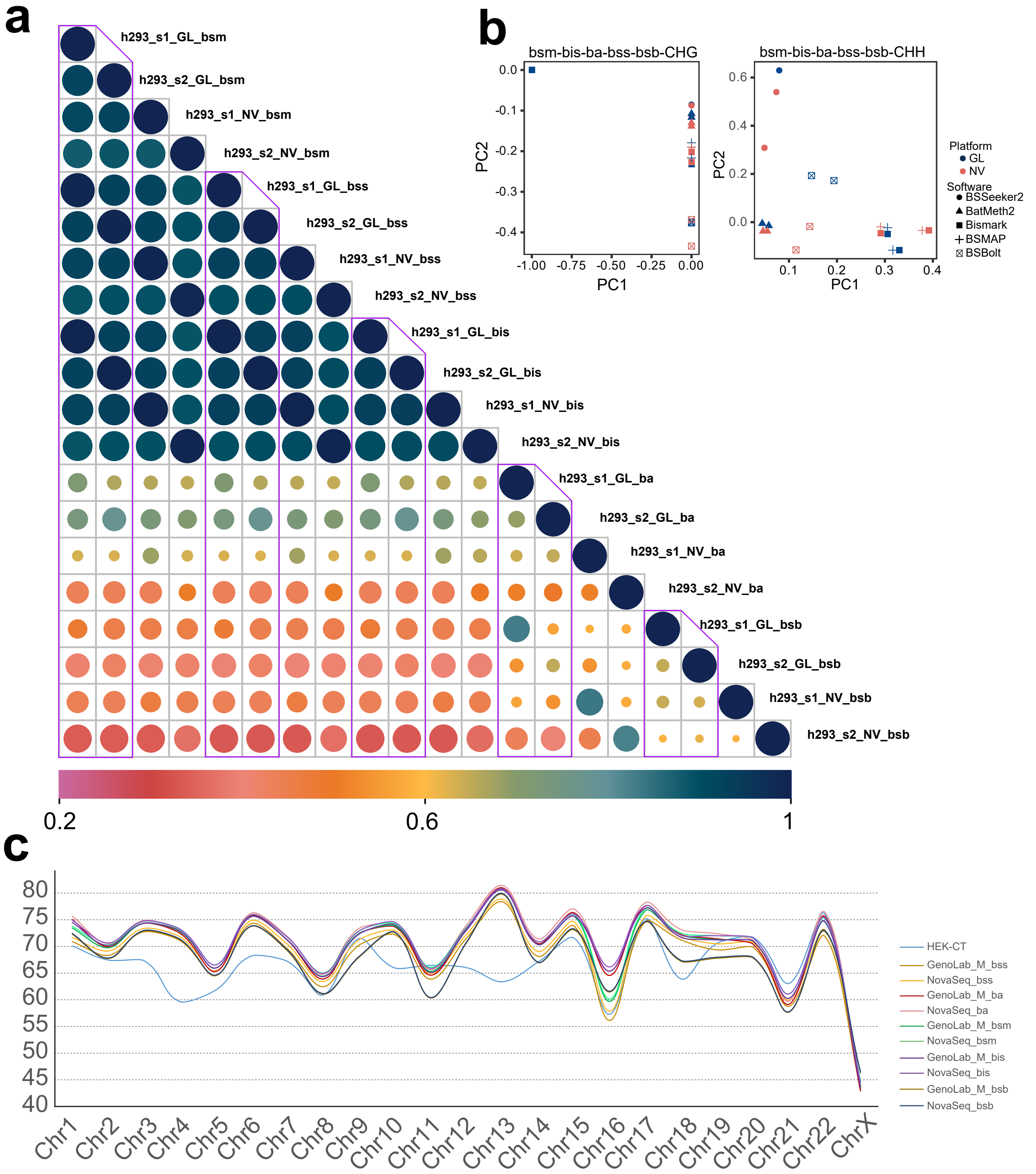

Supplementary Figure 4 The correlationship methylation ratio of chromosomes from different datasets. **a** The correlationship of sample data from four h293 samples and different software. **b** The PCA of methylation ratio of CHG/CHH from different software. **c** The methylation level of different chromosomes from different origins. bsm: BSMAP; bis: Bismark; bss: BS Beeker2; bsb: BSBolt; ba: BatMeth2;
