# Supplementary material for: Systematic and benchmarking studies of pipelines for mammal WGBS data in the novel NGS platform
Source: BMC Bioinformatics. 2023 Jan 31;24:33. doi: 10.1186/s12859-023-05163-w (PMC9890740; doi:10.1186/s12859-023-05163-w)
Supplement: Supplementary file 9 — Additional file 9: Fig S5. The methylation pattern of GNB1 genebody 40 loci. [file 12859_2023_5163_MOESM9_ESM.pdf]

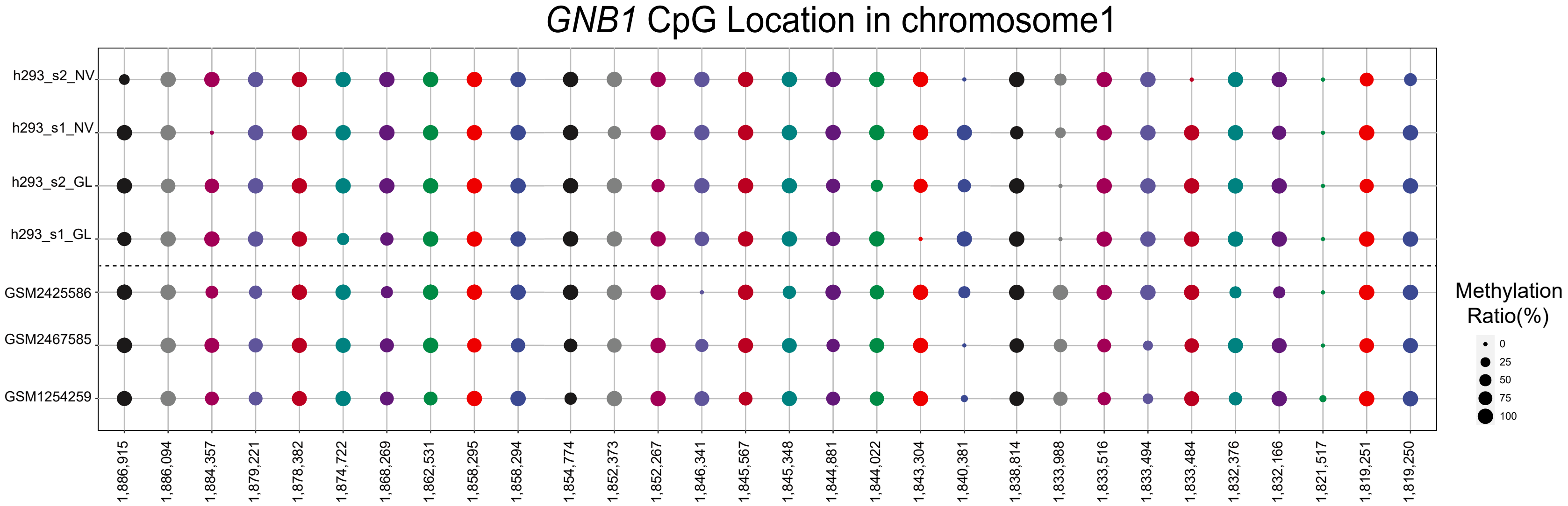

Supplementary Figure 5 The methylation pattern of GNB1 genebody 40 loci.
